# Comparative molecular dynamics simulations of pathogenic and non-pathogenic huntingtin protein monomers and dimers

**DOI:** 10.3389/fmolb.2023.1143353

**Published:** 2023-04-10

**Authors:** Mohammed Khaled, Birgit Strodel, Abdallah Sayyed-Ahmad

**Affiliations:** ^1^ Institute of Biological Information Processing (IBI-7: Structural Biochemistry), Forschungszentrum Jülich, Jülich, Germany; ^2^ Institute of Theoretical and Computational Chemistry, Heinrich Heine University Düsseldorf, Düsseldorf, Germany; ^3^ Department of Physics, Birzeit University, Birzeit, Palestine

**Keywords:** polyglutamine, huntingtin, molecular dynamics, oligomer, aggregation

## Abstract

Polyglutamine expansion at the N-terminus of the huntingtin protein exon 1 (Htt-ex1) is closely associated with a number of neurodegenerative diseases, which result from the aggregation of the increased polyQ repeat. However, the underlying structures and aggregation mechanism are still poorly understood. We performed microsecond-long all-atom molecular dynamics simulations to study the folding and dimerization of Htt-ex1 (about 100 residues) with non-pathogenic and pathogenic polyQ lengths, and uncovered substantial differences. The non-pathogenic monomer adopts a long *α*-helix that includes most of the polyQ residues, which forms the interaction interface for dimerization, and a PPII-turn-PPII motif in the proline-rich region. In the pathogenic monomer, the polyQ region is disordered, leading to compact structures with many intra-protein interactions and the formation of short *β*-sheets. Dimerization can proceed *via* different modes, where those involving the N-terminal headpiece bury more hydrophobic residues and are thus more stable. Moreover, in the pathogenic Htt-ex1 dimers the proline-rich region interacts with the polyQ region, which slows the formation of *β*-sheets.

## 1 Introduction

Huntington’s disease (HD) is an inherited neurodegenerative disease caused by an abnormal expansion in the polyglutamine (polyQ) tract of the N-terminal Huntingtin (Htt-ex1) protein. The elongated polyQ tract mutation is caused by the expansions of nucleotide CAG repeats in exon-1 of the HD gene that encodes the elongated polyQ tract within the Htt-ex1 protein. Furthermore, the expansion of polyQ tracts and their toxicity are correlated to the age of the onset of HD (MacDonald et al., 1993). In HD, Htt-ex1 becomes pathogenic beyond a threshold of 36 glutamine repeats (Takeuchi and Nagai, 2017). The full-length Htt-ex1 is over 3,100 amino acids expressed in all mammalian cells including nerve cells in the brain where it has higher concentrations. Htt-ex1 interacts with a wide range of proteins involved in many cellular processes (Gusella and MacDonald, 2000; Li and Li, 2004; Saudou and Humbert, 2016), but the exact structure and function of Htt-ex1 are still poorly understood (Mangiarini et al., 1996). Nevertheless, HD onset and progression are associated with the misfolding of Htt-ex1 which eventually forms amyloid aggregates that are connected to neuronal cell death (Perutz et al., 1994). Toxic aggregation of Htt-ex1 into amyloid cannot only be observed *in vivo*, but can also be reproduced *in vitro* (Nagai et al., 2007). Several hypotheses have been suggested to explain the aggregation behavior and thereby the toxicity of Htt-ex1. One of these hypotheses proposes that pathogenic Htt-ex1 accumulates into insoluble aggregates in neurons as amyloid fibrillar structures (Miller et al., 2011). Other hypotheses suggest that Htt-ex1 monomers or oligomers with extended polyQ tracts interact with other cellular proteins and alter their functions, which leads to neuronal cell death (Williams and Paulson, 2008; Gkekas et al., 2021).

Importantly, the expansion of the polyQ tract is also associated with many other inherited neurodegenerative diseases (Williams and Paulson, 2008). Thus, several studies have been conducted to investigate the structures and aggregation mechanisms of isolated polyQ peptides. Experiments under different solutions conditions found that isolated polyQ tracts could sample various conformations as collapsed structures (Crick et al., 2006) with random coils (Chen et al., 2001; Klein et al., 2007), *α*-helix (Bhattacharyya et al., 2006), *β*-sheets (Nagai et al., 2007; Darnell et al., 2009) and PPII helix (Chellgren et al., 2006; Darnell et al., 2007; Darnell et al., 2009). The conformational flexibility of polyQ tracts has also been confirmed by computational modeling using molecular dynamics (MD) simulations, as was comprehensively reviewed by Moldovean and Chis (Moldovean and Chiş, 2019). Of particular note are the studies (Długosz and Trylska, 2011; Vöpel et al., 2017; Priya and Gromiha, 2019) as they did not only simulate isolated polyQ stretches as done in the majority of the other simulation studies, but included the first 17 N-terminal residues directly preceding the polyQ sequence and some of the following C-terminal residues. Despite the various experimental and simulation efforts, there is no consensus yet on the preferred polyQ structure in solution. Almost all secondary structures have been suggested, ranging from *α*-helical structures (Elena-Real et al., 2022) to coil and *β*-sheets (Moldovean and Chiş, 2019). Nonetheless, there is ample evidence that indicates that longer polyQ increases the *β*-sheet propensity and aggregation rates (Thakur and Wetzel, 2002; Klein et al., 2007; Sivanandam et al., 2011).

**FIGURE 1:**
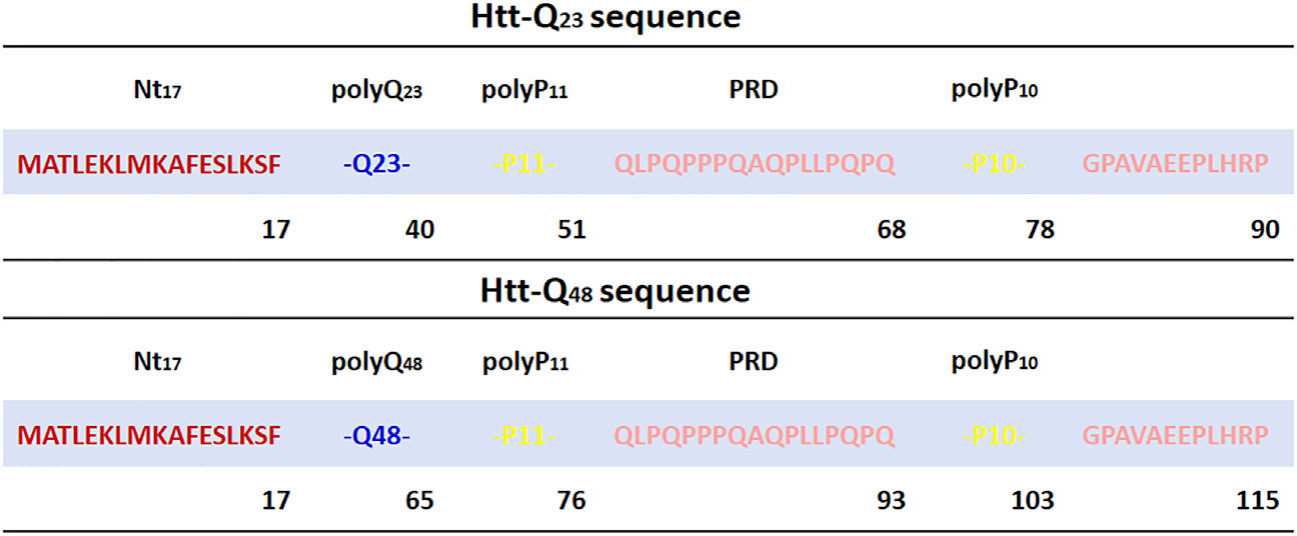
Sequence of Htt-Q_23_ and Htt-Q_48_ studied in this work. The Nt_17_ region is highlighted in red, polyQ in blue, and the PRD is shown in yellow for polyP_11_ and polyP_10_ and rose for the other PRD parts.

The Htt-ex1 consists of three regions (Figure 1): the N-terminal 17 amino acid region (Nt_17_), the polyQ tract region, followed by a proline-rich region (PRD). The N-terminal and proline-rich regions surrounding the polyQ region have critical roles in modulating the aggregation mechanism as has been illustrated in computational and experimental studies (Chow et al., 2012; Qin et al., 2004; Duennwald et al., 2006a; Duennwald et al., 2006b). The Nt_17_ region is known to accelerate the aggregation by forming prefibrillar spherical oligomers with Nt_17_ at their core (Tam et al., 2009; Thakur et al., 2009). Experimentally determined structures of Nt_17_ suggested that it folds into an amphipathic *α*-helix (Kim et al., 2009; Kim, 2013; Michalek et al., 2013). In addition, it has been suggested that the *α*-helix Nt_17_ may initialize the Htt-ex1 aggregation by forming *α*-helix rich oligomers (Hoop et al., 2014; Sahoo et al., 2014; Pandey et al., 2018). In general, it appears that Nt_17_ is mostly helical in Htt-ex1 aggregates (Sivanandam et al., 2011; Jayaraman et al., 2012; Hoop et al., 2014). However, nuclear magnetic resonance (NMR) spectroscopy showed that structures of Nt_17_ are intrinsically disordered and adopt different conformations (Thakur et al., 2009). The PRD, on the other hand, is found to decrease the stability and rate of amyloid-like aggregation without changing the fundamental mechanism of aggregation (Bhattacharyya et al., 2006). NMR and electron paramagnetic resonance (EPR) spectroscopy showed that the PRD tends to adopt similar structures with rich PPII helical structures in both monomers and fibril structures (Bugg et al., 2012; Isas et al., 2015).

Proteins with glutamine repeats are structurally unstable. This makes it difficult to resolve the structure of regions surrounding the polyQ tracts (Takeuchi and Nagai, 2017). As of today, only structures of the Htt-ex1 containing 17 glutamine repeats were resolved by X-ray crystallography (Kim et al., 2009). Thus, various computational studies of model Htt-ex1 have been conducted to illustrate the effect of polyQ tract length on protein structure (Moldovean and Chiş, 2019). Other studies provided insights into the effects of Htt-ex1 flanking domains on its structure (Długosz and Trylska, 2011; Vöpel et al., 2017; Priya and Gromiha, 2019). By adding or removing these domains, the PRD was found to destabilize the protein and inhibited *β*-sheet formation (Lakhani et al., 2010; Williamson et al., 2010). Kang and coworkers carried out MD simulations of full-length Htt-ex1 monomers with different polyQ lengths. Their results suggest a positive correlation between polyQ tract length and the *β*-sheet content (Kang et al., 2017). Finally, the polyQ region was found to be the main driver for Htt-ex1 aggregation (Williamson et al., 2010).

In this work, we performed all-atom MD simulations to investigate the structural properties of Htt-Q_*n*_ monomers and dimers with *n* = 23 for non-pathogenic Htt-ex1 and *n* = 48 for disease-causing Htt-ex1. In addition to including the Nt_17_ region, we also added 50 amino acids to model the impact of the PRD on the polyQ region (Figure 1). With this, our simulated systems are longer than those usually simulated. In addition, we used the Charmm36m force field, which has been adapted to proteins with low-complexity sequences (Huang et al., 2017) and has been shown to be useful in predicting the conformational ensemble of intrinsically disordered proteins (Robustelli et al., 2018; Paul et al., 2021) and amyloid aggregates (Samantray et al., 2020), but has not yet been applied to Htt-ex1 proteins. Furthermore, we performed simulations on the microsecond time scale, which is longer than in most preceding simulations. Our study therefore provides updated insights into the combined effects of Nt_17_, PRD, and polyQ tract length on the Htt-ex1 structure and aggregation.

## 2 Materials and methods

### 2.1 Initial structures and system preparations

The two simulated sequences are MATLEKLMKAFESLKSF-Q_*n*_-P_11_-QLPQPPPQAQPLLPQPQ-P_10_-GPAVAEEPLHRP with *n* = 23 and *n* = 48 glutamine residues (Figure 1). The X-ray crystal structure of Htt-ex1 (PDB ID: 3IOT) (Kim et al., 2009) consists of an *α*-helix for Nt_17_ and the *α*-helix extends up to 15 glutamines into the polyQ_17_ tract. For the polyP_11_ region, extended loops, random coil conformations and also PPII-helix formation were observed. However, since the structure of the full-length Htt-ex1 protein is not completely resolved, the initial structures were built using the Avogadro software (Hanwell et al., 2012) and PyMOL (Schrödinger, 2022) by adding the peptide fragment Q_11_-PRD and Q_36_-PRD to the N-terminal part Nt_17_-Q_12_ taken from the PDB structure 3IOT for modeling the Htt-Q_23_ and Htt-Q_48_, respectively. This resulted in extended structures for both proteins, which were solvated with water and then collapsed through initial 20 ns MD simulations to reduce the system size in the production MD simulations.

### 2.2 MD simulations

All MD simulations were carried out using GROMACS 2020 (Van Der Spoel et al., 2005; Abraham et al., 2015) as MD program, employing the atomistic force field Charmm36m along with the Charmm-modified TIP3P water model (Huang et al., 2017). The initial (already collapsed) monomer structures were solvated in a ∼2,000 nm^3^ cubic box, including 150 mM Na^+^ and Cl^−^ ions and overall system neutralization, leading to about 200,000 atoms. The energy of each system was initially minimized using the steepest descent algorithm (Müller and Brown, 1979; Zhang, 2015), followed by 1.0 ns equilibration and then the production runs under NPT ensemble conditions. The pressure and temperature were maintained at 1.0 bar and 298 K using the Parrinello-Rahman pressure coupling method (Parrinello and Rahman, 1981; Parrinello and Rahman, 1982) and a velocity-rescale thermostat method (Bussi et al., 2007), respectively. All simulations implied periodic boundary conditions applied in all directions and using a cutoff distance of 1.2 nm for the calculation of the non-bonded interactions in real space. The electrostatic interactions were computed using the particle mesh Ewald (PME) method (Essmann et al., 1995). The LINCS algorithm was applied (Hess et al., 1997) to constrain bond lengths, and a leapfrog integrator (Van Gunsteren and Berendsen, 1988) was used to integrate the equations of motions with a 2 fs time step. For all simulations it was checked that there were no self-interactions of the proteins with their periodic images during the course of the simulations as a result of conformational reorientations.

For each monomer system, 5 × 1.0 *μ*s simulations were carried out. Simulation 2 was initiated from the final snapshot of the first simulation, while simulations 3 through 5 were started from the central structure of the most populated conformational cluster of the respective preceding simulation. For the analysis, the first simulation per sequence was discarded due to the bias of the extended initial structures, despite the initial 20 ns MD simulation to collapse these structures. This bias is visible in the time traces of various observables (Supplementary Figures S1–S3). To assesses the convergence of the monomer simulations, the autocorrelation functions of various structural properties were calculated for the individual 1.0 *μ*s runs (Supplementary Figure S4). They all tend to zero within 500 ns, indicating loss of memory from initial structures. Nonetheless, while general convergence of the individual trajectories is found, certain fluctuations of the various structural properties persist, which is an expected behavior for intrinsically disordered proteins (Paul et al., 2021). We further conducted the Augmented Dickey Fuller (ADF) test as a unit-root test to assess the stationarity of the monomer data. The ADF test was applied to the concatenated time series of various observables (Supplementary Table S1). The obtained *p*-values for all the tested observables are below 0.05, and the test statistics are also smaller than the critical values, thereby rejecting the null hypothesis and indicating that the data are stationary.

For both proteins, 3 × 2.0 *μ*s dimer simulations were carried out in a cubic simulation box with a volume of about 1,925 nm^3^ and a total number of 190,000 atoms. The six most dominant conformational clusters determined from the previous monomer simulations were used as starting structures for the monomers in the dimer simulations. The simulations were initiated by randomly orienting two monomers in the simulation box, ensuring a minimum distance of 5 nm between them.

### 2.3 Analysis

The MD simulations were analyzed using different tools, which were invoked from GROMACS or from the MDAnalysis Python package (Michaud-Agrawal et al., 2011; Gowers et al., 2016). For the analysis of the monomers, the 4 × 1.0 *μ*s simulations per protein (ignoring the initial simulation) were concatenated. In the case of the dimers, the 3 × 2.0 *μ*s simulations per protein were joined for analysis. The determination of the secondary structure was done with the DSSP (Define Secondary Structure of Proteins) program (Kabsch and Sander, 1983) as available *via* GROMACS. The PPII-helix structures were determined based on the dihedral angles of the protein backbone, *ϕ* and *ψ*, which fall into the range of −104 ≤ *ϕ* ≤ −46 and 116 ≤ *ψ* ≤ 174 for a PPII helix (Mansiaux et al., 2011; Yu et al., 2021). Previous studies have demonstrated that the assignment of the PPII helix using either DSSP-PPII or the backbone dihedral-angles approach provide highly similar results (Jephthah et al., 2021; McIvor et al., 2022). Conformational clustering of the trajectories was accomplished using the algorithm by Daura et al. (Daura et al., 1999), which is a nearest neighbor algorithm, using the root mean square deviation (RMSD) between all trajectory snapshots together with an RMSD cutoff of 0.5 nm to assign the neighbors. For the RMSD calculations, only the C*α* atoms were used for both the alignment and actual calculation. To determine the contacts between residues, we calculated minimum distances for all residue pairs, within the proteins and also between the proteins in the case of the dimers. The time-averaged distances between the residues are presented as distance matrices. The contacts were further analyzed based on their interaction type, i.e., hydrophobic, H-bond, or salt-bridge interactions, using the CONAN software (Mercadante et al., 2018). This tool was also employed to identify correlations in the motions of the proteins in their monomeric form. Further analysis involved the calculation of the radius of gyration (*R*_*g*_) of the proteins, the distance between the C*α* atoms of the protein termini (denoted as end-to-end distance or *d*_*ee*_), and the root mean square fluctuations (RMSFs) of the C*α* atoms after alignment to the time-averaged structure. The free energy as a function of *R*_*g*_ and *d*_*ee*_ was calculated as Δ*G*(*R*_*g*_, *d*_*ee*_) = −*k*_*B*_ *T*[ln *P*(*R*_*g*_, *d*_*ee*_) − ln *P*_max_(*R*_*g*_, *d*_*ee*_)], where *k*_*B*_ is the Boltzmann constant, *T* is room temperature, *P*(*R*_*g*_, *d*_*ee*_) is the probability of the protein to have given values (*R*_*g*_, *d*_*ee*_), and *P*_max_(*R*_*g*_, *d*_*ee*_) is the maximum of that probability distribution. The free energy was also determined as a function of the first two principal components, PC1 and PC2, that were determined from a principal component analysis (PCA) using the Cartesian coordinates of the C*α* atoms.

The solvent-accessible surface area (SASA) was calculated for both the whole proteins and selected residues. The SASA of the proteins was further distinguished into the hydrophobic and polar solvent-accessible surface areas using the residue sets (Met, Ala, Phe, Leu, Pro, Gly, Val) and (Asp, Glu, His, Lys, Arg, Ser, Thr, Gln), respectively. The standard errors of the SASA values were determined *via* block averaging with 4 blocks, each containing 1,000 data points.

## 3 Results and discussion

### 3.1 The pathogenic Htt-ex1 monomer is more compact and has a higher *β*-sheet propensity than its non-pathogenic counterpart

In this section, we illustrate the structural differences between Htt-Q_23_ and Htt-Q_48_ monomers by the analysis of various structural properties, including the RMSD, *R*_*g*_, *d*_*ee*_, secondary structure, and the SASA. The evolution of these structural properties are presented in Supplementary Figures S1–S3.

#### 3.1.1 Secondary structure and structural flexibility

**FIGURE 2:**
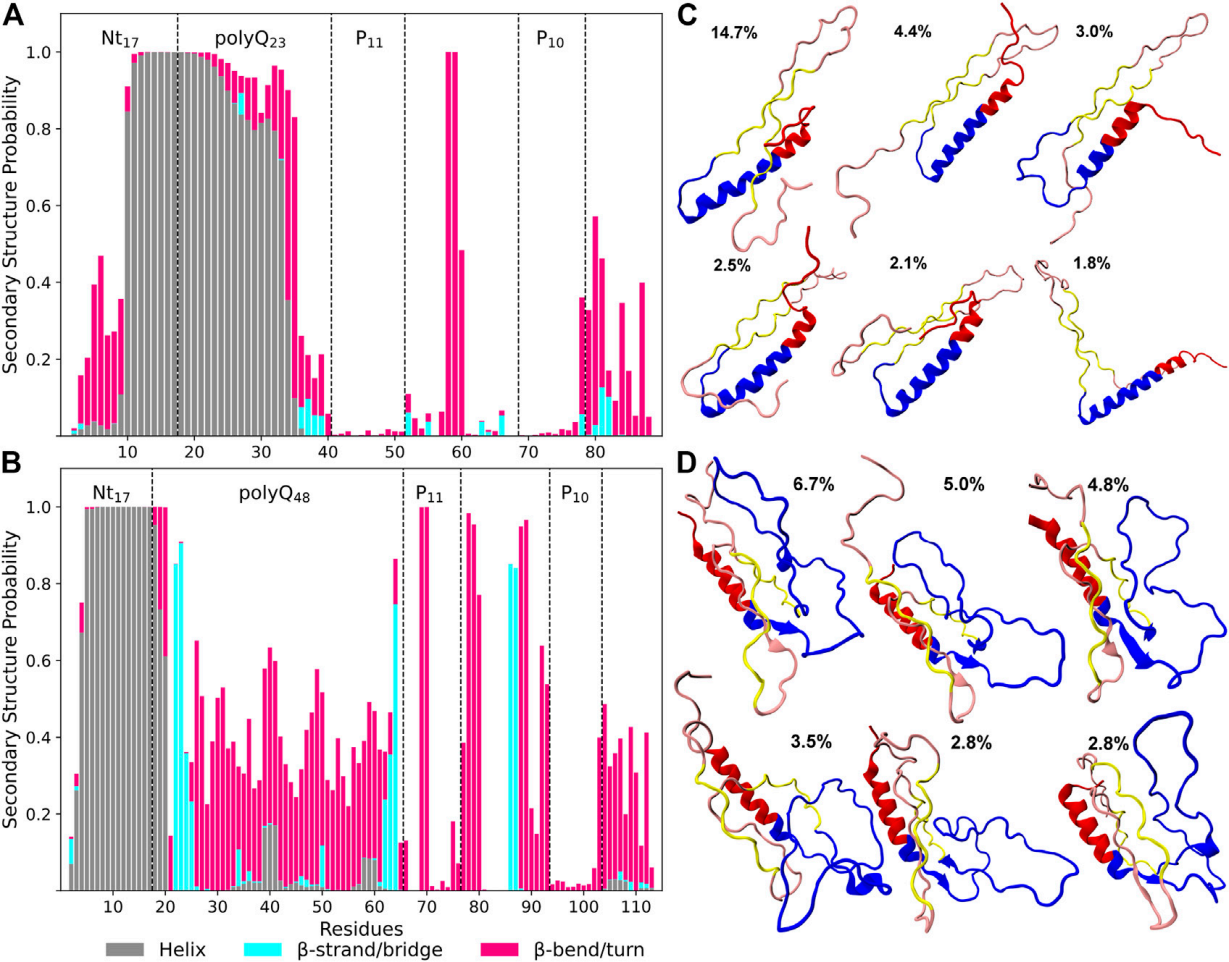
Secondary structure preferences per residue of Htt-Q_23_ **(A)** and Htt-Q_48_ **(B)** monomers. The bars represent the additive secondary structure probabilities consisting of *α*-helix (gray), *β*-strand/bridge (cyan), and *β*-turn/bend (magenta). The difference to 1.0 presents the other structures, in particular PPII and random coil. Representative structures were determined from conformational clustering using an RMSD cutoff of 0.5 nm for Htt-Q_23_ **(C)** and Htt-Q_48_ **(D)**. The population of each cluster is given. The Nt_17_ region is shown in red, polyQ in blue, polyP_11_ and polyP_10_ in yellow, and the rest of the PRD in rose.

Figures 2A, B indicate significant differences in the secondary structure profiles of the two proteins. Htt-Q_48_ has a greater tendency to adopt *β*-sheet, bend and turn conformations, while Htt-Q_23_ seems to favor *α*-helix and random coil conformations. The first ten residues of the Nt_17_ region of Htt-Q_48_ form a stable *α*-helix, which is contrary to Htt-Q_23_ where these N-terminal residues prefer disordered structures with some bends and turns. However, the following residues of Htt-Q_23_ form an *α*-helix that extends up to 16 glutamines into the polyQ region. The last five residues of polyQ_23_ are mostly disordered with a negligible *β*-sheet content between residues 36–39. The polyP regions of Htt-Q_23_ adopt straight PPII helical structures, which is revealed by an analysis of the Ramachandran angles (Supplementary Figure S5). To further evaluate the propensities of the individual residues to adopt a PPII helix, we calculated the backbone dihedral angles *ϕ* and *ψ* and used them to calulate the PPII-helix probabilities (Supplementary Figure S6). These probabilities confirm a high propensity of PPII-helix formation in polyP_11_ and polyP_10_ of both proteins. In particular, the average *ϕ* and *ψ* angles are very similar for the residues of these regions. A rather high probability of PPII-helix formation is also found for most of the other residues of the PRD of both Htt-Q_23_ and Htt-Q_48_, yet with a higher number of kinks and turns present in the PRD of Htt-Q_48_. The *α*-helical regions encompassing the second half of Nt_17_ and most of the polyQ region of Htt-Q_23_ and most of the Nt_17_ region of Htt-Q_48_ have zero propensity to adopt PPII-helix structures. This suggests a more disordered polyQ region in Htt-Q_48_ than in Htt-Q_23_, which in the former can also adopt *ϕ* and *ψ* angles that fall within the PPII-helix region with an average probability of 44% (Supplementary Figure S6).

These observations are consistent with previous NMR solution results which showed that the wild-type Huntingtin protein Nt_17_ has a propensity to adopt helical structures that extended to the polyQ domains (Baias et al., 2017; Newcombe et al., 2018). The N-terminal helix in Htt-Q_48_ is mainly limited to the Nt_17_ region, while the majority of its polyQ region forms a random coil or a bend/turn conformation. However, the terminal residues of the extended polyQ region shows a high propensity to adopt *β*-sheet structures. This higher *β*-sheet content compared to Htt-Q_23_ is consistent with previous experimental and MD simulation results (Nagai et al., 2007; Heck et al., 2014; Kang et al., 2017). These studies suggested that the *β*-sheet content within the polyQ region is associated with longer polyQ lengths and may play a role in amyloid fibril formation (Nagai et al., 2007; Kang et al., 2017). Experiments with synthesized *β*-sheet in the polyQ domains showed an increased rate of aggregation (Kar et al., 2013). This suggests that the *β*-sheet conformations within the polyQ region represent the aggregation-prone structures giving rise to amyloid fibrils (Kar et al., 2013).

We continued with a structural cluster analysis to see how the different secondary structures are arranged in the proteins. The six most populated clusters per protein are shown in Figures 2C, D. Five of the shown cluster structures of Htt-Q_48_ have *β*-sheet conformations that involve polyQ regions (residues 22–25 and 62–64) and parts of the PRD (residues 86–87), with two or three *β*-strands per sheet. However, in the fourth cluster structure coil conformations outside the helical Nt_17_ region dominate, indicating that the *β*-sheet formation in Htt-Q_48_ is transient. Htt-Q_23_ samples a long helix in the Nt_17_ and most of the polyQ region. The whole protein is less collapsed than Htt-Q_48_, but rather extended, which results from the long N-terminal *α*-helix plus the two polyP stretches in PPII conformation that prefer to align in an antiparallel arrangement in Htt-Q_23_. This PPII-turn-PPII arrangement is also made possible because residues Pro58/Glu59 between polyP_11_ and polyP_10_ allow for a turn to be formed with 100% probability. Apart from that bend, only some further bends and turns are formed in the last ten residues of the PRD of Htt-Q_23_, while in Htt-Q_48_ several residues along the PRD sequence have a high bend/turn propensity. Together with the disordered polyQ region, this gives the longer protein a more collapsed and also more disordered shape. The Htt-Q_23_ results suggest that the region reaching from P_11_ to P_10_ strongly affects the folding of this protein by forming PPII helices that also have an ordering effect on the polyQ region, leading to helix formation in most of the 23 glutamine residues.

**FIGURE 3:**
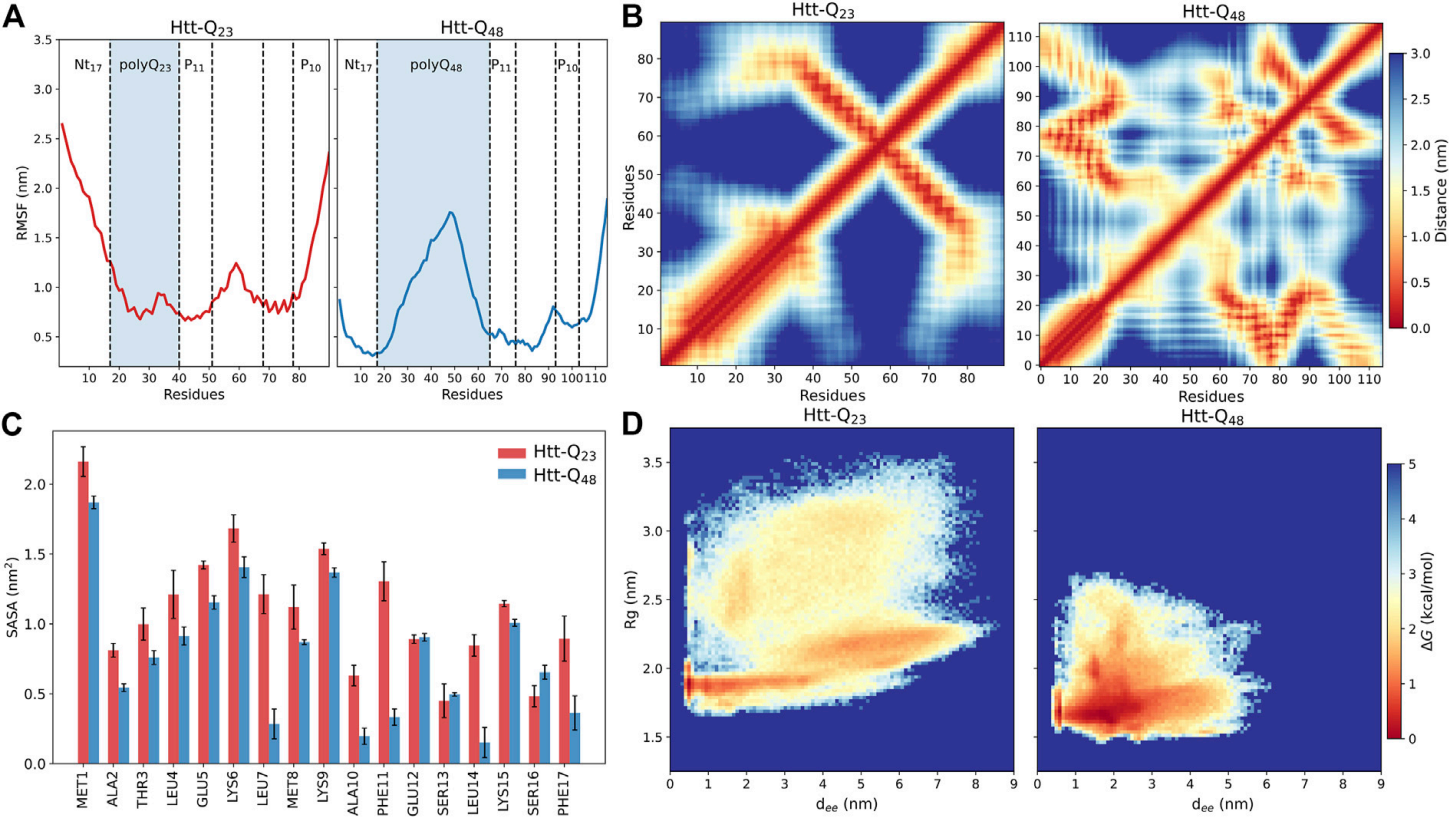
Structural properties of Htt-Q_23_ and Htt-Q_48_ monomers. **(A)** The RMSFs of the C*α* atoms of Htt-Q_23_ (left) and Htt-Q_48_ (right). The polyQ regions are highlighted by a blue shade. **(B)** Intra-protein distance matrices of Htt-Q_23_ (left) and Htt-Q_48_ (right). The color bar on the right shows the average residue-residue distance (in nm). **(C)** The average SASA of the Nt_17_ residues (with standard errors) for Htt-Q_23_ (red) and Htt-Q_48_ (blue). **(D)** The free energy surface of Htt-Q_23_ (left) and Htt-Q_48_ (right) was calculated using the radius of gyration and the end-to-end distance. The color bar shows the value of the free energy (ΔG) in kcal/mol.

The different structural flexibilities of Htt-Q_23_ and Htt-Q_48_ are also visible in the RMSFs of their C*α* atoms (Figure 3A). The RMSFs reveal for Htt-Q_23_ a higher flexibility in the Nt_17_, where the first ten residues did not form a helix as in Htt-Q_48_, while the C-terminal of the PRD region is flexible in both proteins. However, the rest of the Htt-Q_23_ is more stable than Htt-Q_48_. In the latter, the polyQ region is particularly flexible, especially in its middle. These results suggest that longer polyQ tracts stabilize the N-terminal half of Nt_17_, while inducing disorder and flexibility in the rest of the protein.

#### 3.1.2 Intra-protein contacts and solvent accessibility

To further define the structural differences, we calculated the average distances between the protein residues to characterize the coupling between different domains (Figure 3B). The distance maps confirm the tendency of Htt-Q_48_ to adopt more compact structures as indicated by the significant contacts among Htt-Q_48_ residues. These contacts enable *β*-sheet formation in Htt-Q_48_ to take place. The contact map of Htt-Q_23_ is defined by the long *α*-helix in the N-terminal protein part and the alignment between polyP_11_ and polyP_10_ in the C-terminal half. The alignment of the polyP regions in Htt-Q_23_ is stabilized by hydrophobic interactions (Supplementary Figure S7). In addition, the polyQ region sporadically interacts with polyP_10_ and the C-terminal residues (79–90). Conversely, the Nt_17_ of Htt-Q_48_ tends to interact with polyP_11_, the middle of PRD (77–87)—both mediated by hydrophobic interactions –, and the C-terminal (98–110). Moreover, the polyQ region of Htt-Q_48_ can also interact with PRD domains excluding the PRD C-terminal, which involves H-bond formation. Strikingly, the polyP regions interact with different domains of Htt-Q_48_. A remarkable difference between the two proteins is that the Nt_17_ region of Htt-Q_48_ shows strong contacts with PRD regions, which is hardly the case in Htt-Q_23_. The stability of the intra-protein interaction was further assessed by calculating their probability expressed as percent lifetime (Supplementary Figure S8). This analysis reveals that the hydrophobic interactions between the polyP regions in Htt-Q_23_ and those involving Nt_17_ in Htt-Q_48_ are the most stable interactions. The other very stable interactions are the H-bonds in *α*-helices that are present in either protein. The terminal interactions in Htt-Q_48_ seem to stabilize the helix in the first ten Nt_17_ residues, and also induce bends/turns with some helical propensity in the last ten C-terminal residues. It has been shown that the helical structure of Nt_17_ is characterized by amphipathic properties (Kelley et al., 2009; Długosz and Trylska, 2011). Thus, the hydrophobic residues of the Nt_17_ minimize their contact with water by interacting with the hydrophobic polyP_11_ and the following PRD mixed region. The different intra-protein interactions in the two proteins also manifest themselves in different coupling between their domains, as revealed by dynamic cross-correlation maps that uncover correlations in the motions (Supplementary Figure S9). In Htt-Q_23_, all residues spanning from the start of polyP_11_ to the end of polyP_10_ move in a correlated fashion, as they are coupled *via* their strong intra-protein interactions, while this region moves anticorrelated to residues 1–40 containing Nt_17_ and polyQ_23_. In Htt-Q_48_ the correlated and anticorrelated motions are more fragmented along the sequence as a result of the more distributed intra-protein contacts. Most notably, the motions of the polyQ_48_ are mainly anticorrelated to the motions of all other residues on either side of that region.

An analysis of the SASA per residue of Nt_17_ confirms that in Htt-Q_48_ these residues are better shielded from water; this difference is particularly noticeable for the hydrophobic residues Leu4, Leu7, Ala10, Phe11, Leu14, and Phe17 (Figure 3C). This is consistent with previous observations that the Nt_17_, polyP_11_ and mixed region of Htt-ex1 form a hydrophobic core (Williamson et al., 2010; Długosz and Trylska, 2011) and is confirmed by the hydrophobic and polar SASA of the whole proteins. Htt-Q_48_ exposes more polar residues and prefers to bury the hydrophobic ones compared to Htt-Q_23_ (Supplementary Figure S10A). However, the polar SASA results are not directly comparable, as Htt-Q_48_ has 25 more polar residues than Htt-Q_23_. Supplementary Figure S10B shows that the polyQ stretch of Htt-Q_48_ is solvent-exposed, shielding the hydrophobic core from the solvent from one side, while on the other side the shielding is done by the hydrophilic face of the N-terminal helix. In Htt-Q_23_, on the other hand, the hydrophobic residues cluster around the polyP PPII helices and interact with the hydrophobic residues of the Nt_17_ helix (Supplementary Figure S10B), leaving them more solvent-exposed as the 23 glutamine residues are not enough to cover them in that geometry. Nonetheless, this protein structure is stable as the pronounced intra-protein contacts attest. Previous studies suggested that the increase of the interaction surface of the polyQ tract could enhance the protein-protein interactions in the cell by enhancing the binding (Warner et al., 2017; Newcombe et al., 2018), which according to the solvent exposure of the polyQ tract seen here for Htt-Q_48_ seems plausible. Overall, the intra-protein interactions of Htt-ex1 are highly associated with the increase of polyQ length, and are mainly governed by the increased protein flexibility in that region, allowing the formation of a hydrophobic core in Htt-Q_48_.

#### 3.1.3 Conformational variability

To highlight the conformational differences between the two proteins and get better insights into how the elongated polyQ tract affects the conformational energy landscape, we calculated the free energy surface (FES) using the radius of gyration and the end-to-end distance as order parameters. Figure 3D illustrates the structural differences between the two proteins. The FES of Htt-Q_23_ shows that it covers a wider range of the conformational (*R*_*g*_, *d*_*ee*_) space than Htt-Q_48_, which populates regions with more compact structures, confirming our observations made thus far. Htt-Q_23_ prefers conformations with larger *R*_*g*_ values and end-to-end distances due to its elongated shape. Its greater flexibility results mainly from the larger conformational freedom of the first ten residues, that together with the C-terminal motions lead to a large distribution of the *R*_*g*_ and *d*_*ee*_ values in Htt-Q_23_. As a result, there are structures where the two termini are very close to each other (e.g., clusters 1 and 5 in Figure 2C), structures where they are directed away from each other (cluster 2), and the intermediate cases (clusters 3, 4 and 6). Nonetheless, it should be reiterated that the overall fold of Htt-Q_23_ defined by the N-terminal helix and the PPII-turn-PPII motif reaching from polyP_11_ to polyP_10_ is rather stable despite the FES suggesting otherwise. Since *d*_*ee*_ and *R*_*g*_ are partially correlated, although in Htt-Q_23_ small *d*_*ee*_ values are possible for large *R*_*g*_ and the converse is also true, i.e., small *R*_*g*_ with large *d*_*ee*_ values, we also created FESs as a function of the two main pincipal (orthogonal) components, PC1 and PC2 (Supplementary Figure S11A). These suggest that the two proteins cover an equally large conformational space. However, the two FESs cannot be directly compared because the principal components of Htt-Q_23_ and Htt-Q_48_ are not identical, as the two proteins have different sizes. Projection of the individual trajectories onto PC1-PC2 space (Supplementary Figure S11B) shows that Htt-Q_48_ tends toward more compact structures with increasing simulation time, whereas the shorter protein continues to explore the entire conformational space. In summary, the main conclusion from the FES analysis is that Htt-Q_48_ prefers to form more collapsed structures, which is in agreement with its numerous intra-protein contacts. Several experiments and simulations studies suggested that the polyQ tracts prefer to adopt collapsed conformations due to their poor water solubility (Kang et al., 2017). However, this only partly agrees to our findings as we find the long polyQ tract of Htt-Q_48_ to shield the more hydrophobic residues from the water, giving rise to more collapsed conformations than on average seen for Htt-Q_23_ where the polyQ tract is not long enough to provide sufficient shielding and forms a helix instead.

### 3.2 Non-pathogenic and pathogenic Htt-ex1 dimerize in distinct patterns

We investigated the dimerization between the Htt-ex1 monomers to obtain information on the first oligomerization step. For either protein, we observed an association between the monomers within ∼100 ns of the simulation time (Supplementary Figure S12). The dimerization modes are analyzed in more detail in the following.

#### 3.2.1 Interaction interfaces and dimer structures

**FIGURE 4:**
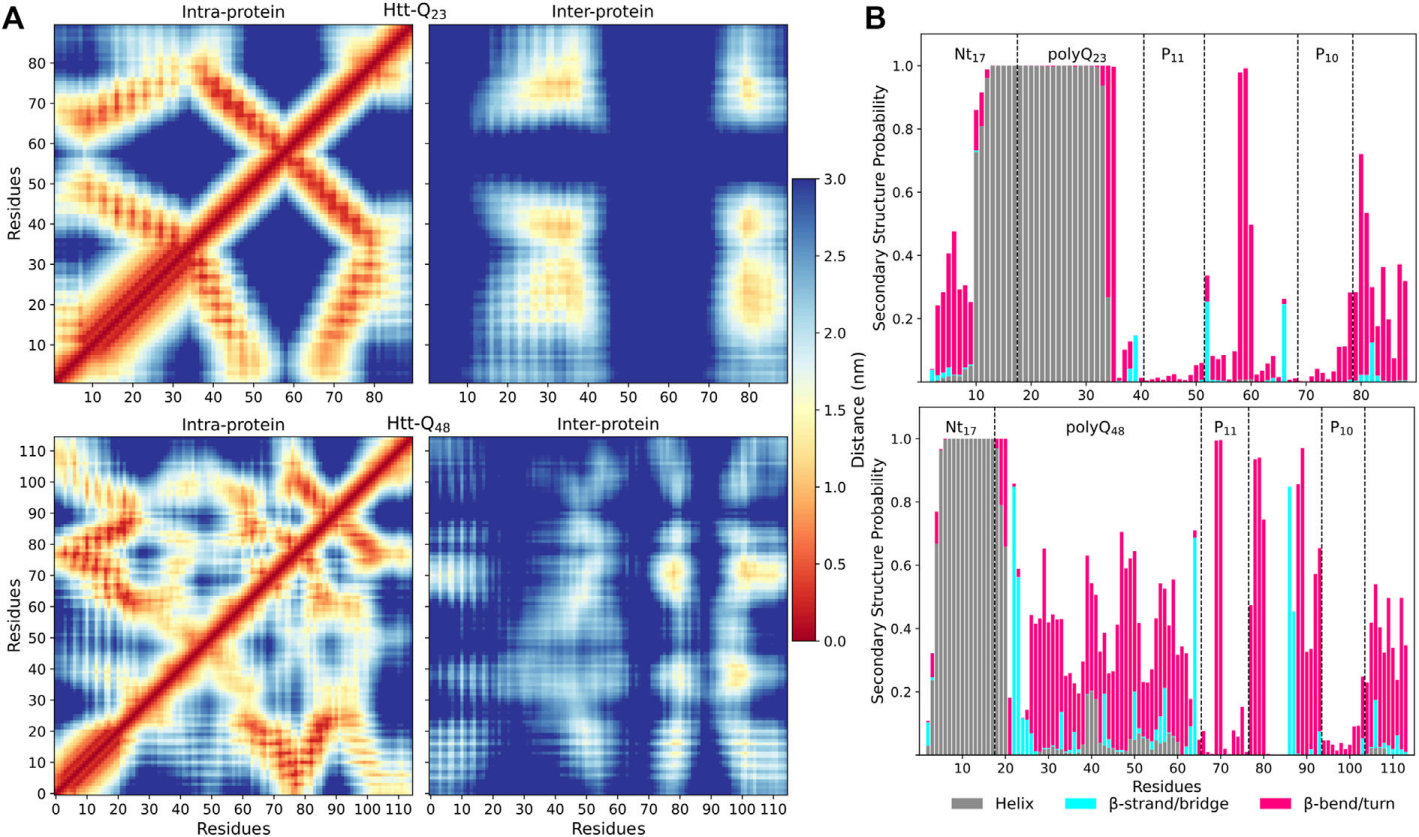
Residue-residue contacts and secondary structures in Htt-Q_23_ and Htt-Q_48_ dimers. **(A)** The intra- and inter-protein contacts between residues for Htt-Q_23_ (top) and Htt-Q_48_ (bottom) dimers. The intra-protein contacts within protein 1 are shown below the main diagonal and within protein 2 above the main diagonal. The color bar on the right shows the average distances (in nm). **(B)** The time- and protein-averaged probability of secondary structure per residue for Htt-Q_23_ (top) and Htt-Q_48_ (bottom) dimers. The bars represent the additive secondary structure probabilities consisting of *α*-helix (gray), *β*-strand/bridge (cyan), and *β*-turn/bend (magenta). The difference to 1.0 presents the other structures, in particular PPII and random coil.

To understand how the proteins interact with each other, we calculated intra- and inter-protein residue-residue distance matrices (Figure 4A). They reveal different interaction patterns within the Htt-Q_23_ and Htt-Q_48_ dimers. In Htt-Q_23_, there are four pronounced interaction regions between the two proteins: i) polyQ/polyP_11_ with polyQ/polyP_11_, ii) polyQ/polyP_11_ with polyP_10_/PRD (79–90), iii) polyP_10_/PRD(79–90) with polyQ/plolyP_11_, iv)polyP_10_/PRD(79–90) with ployP_10_/PRD(79–90). Nt_17_ and PRD(52–68) are not involved at all in the dimerization of Htt-Q_23_. Htt-Q_48_, on the other hand, features intermolecular interactions across the whole sequence, which are, however, less clear-cut. This indicates that different dimer configurations are possible for Htt-Q_48_. The most prominent interactions involve the polyP regions, polyP with mixed regions in PRD, and polyP with polyQ. Furthermore, the polyQ is seen to interact with the whole protein, while the Nt_17_ shows interactions with polyP_11_, polyQ, and the C-terminal of the PRD.

**FIGURE 5:**
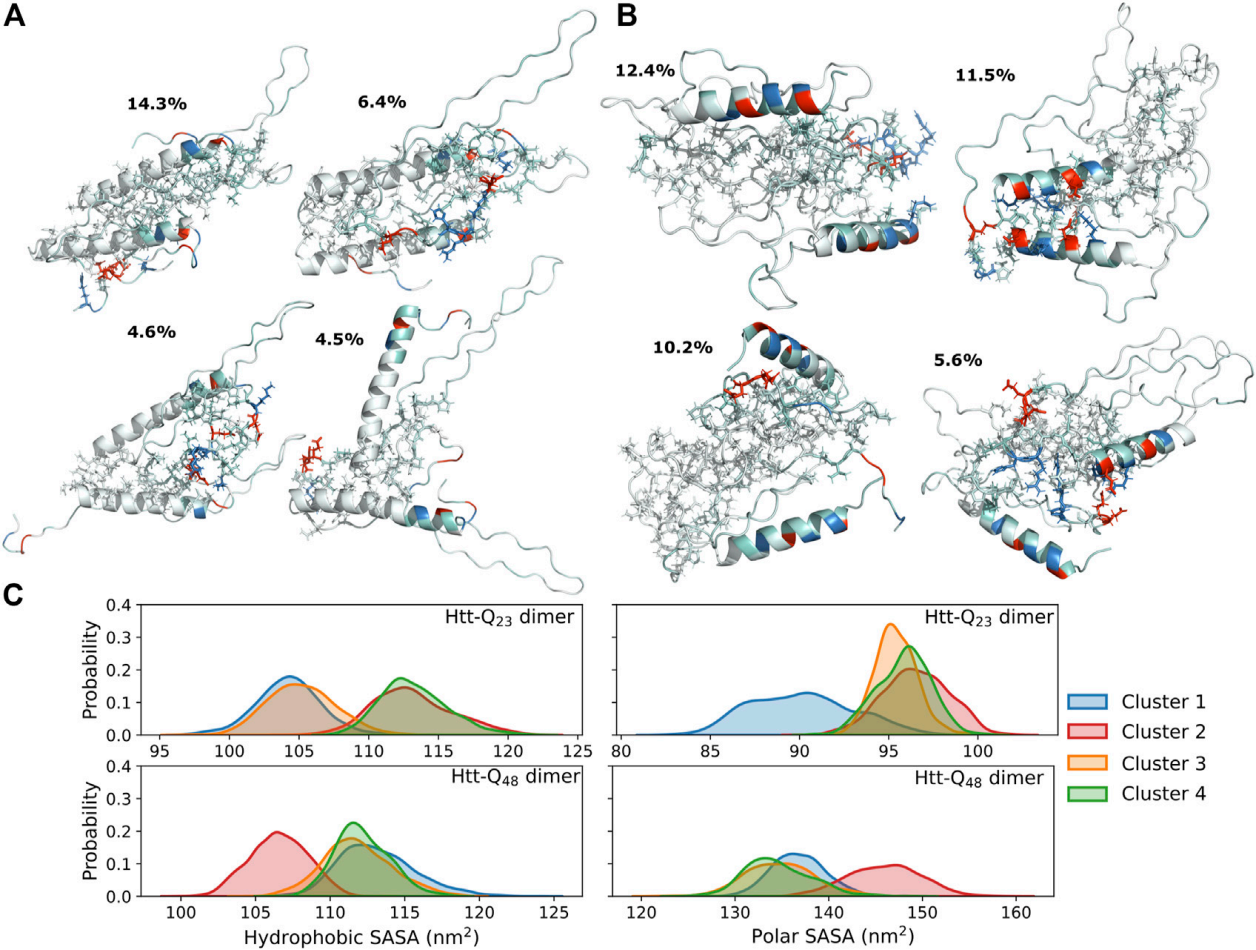
Structures and SASAs of dimers. The four most populated cluster structures for the Htt-Q_23_ **(A)** and Htt-Q_48_ **(B)** dimer. The proteins are shown as cartoons and colored red for the negatively charged residues, blue for the positively charged ones, and white otherwise. The side chains at the protein interfaces are shown as sticks. **(C)** The distribution of the hydrophobic (right) and polar (left) SASAs of the four most populated clusters for Htt-Q_23_ (top) and Htt-Q_48_ (bottom) dimers are shown. The results for the four clusters are shown in different colors according to the color mapping on the right.

The intra-protein interactions in the two proteins of the Htt-Q_48_ dimers are very similar to the ones found for the corresponding monomer (Figure 3B). This implies that the aggregation did not cause major structural transitions, which is confirmed by the secondary structure analysis (Figure 4B vs. Figure 2A). A small difference in Htt-Q_23_ is that dimerization stabilized the Nt_17_/polyQ helix, while in Htt-Q_48_ a small increase in the tendency for *β*-sheet formation is observed across the polyQ region. For more *β*-sheet formation as a result of Htt-Q_48_ dimerization to occur, we expect that more simulation time than the 6 × 2 *μ*s sampled here is required. In Htt-Q_23_, in addition to the intra-protein contacts that were already present in the monomer, two more contact areas within the proteins emerged: the Nt_17_ and first half of the polyQ region interacts with both polyP_11_ and polyP_10_. This speaks for small reorientations of the secondary-structure elements with respect to each other. In order to further understand the interaction interfaces between the proteins, we performed structural clustering and analyzed the hydrophobic and the polar SASAs of the dominant dimer clusters. Figures 5A, B show the representative structures of the four most populated clusters. In general, the interaction interfaces between the Htt-Q_23_ proteins are mainly governed by the interactions of the partly amphipathic Nt_17_/polyQ helices. The two helices tend to be in a parallel orientation to each other, and their interactions add helical stability. The polyP regions, on the other hand, are hardly involved in the interaction interface and instead point away from it. On the other hand, the Htt-Q_48_ dimer clusters reveal a more diverse aggregation behavior with different interaction interfaces where the polyP and the PRD take part in them.

#### 3.2.2 Comparison with experimental observations

Our observations are in agreement with atomic force microscopy (AFM) results that identified oligomeric aggregates of Htt-Q_20_ to be structurally different from those of Htt-ex1 with disease-causing polyQ lengths (Legleiter et al., 2010). The Htt-Q_20_ oligomers did also not transit to form fibril structures. In another study it was shown that the addition of isolated polyP to Htt-ex1 could block the initial aggregation by interacting with Nt_17_ and reduce the ability of headpiece dimerization (Arndt et al., 2019). This speaks for the general involvement of Nt_17_ in the aggregation of Htt-ex1, which is also seen here for both Htt-Q_23_ and Htt-Q_48_. Another possible aggregation-modifying effect of added polyP is that it can also interact with the polyQ region and stabilize it by promoting it to form PPII helix (Darnell et al., 2007; Darnell et al., 2009), which could prevent the nucleation in the oligomers or promote aggregation pathways that are not mediated by the Nt_17_ headpieces (Arndt et al., 2019). Pandey et al. proposed that the Htt-ex1 aggregation is initiated *via* the Nt_17_ headpiece, suggesting that the aggregation pathway of synthetic Htt-ex1 protein into oligomers starts with the self-assembly into an *α*-helix rich oligomer with helical Nt_17_ in the core, followed by *β*-sheet formation within the polyQ tracts. The final and slowest step is the structural maturation of the PRD (Pandey et al., 2018). Through the simulations performed here, we were able to capture the event of Nt_17_ headpiece interactions for Htt-Q_48_ (Figure 5B, cluster 2). Dimerization *via* the Nt_17_ helices results in a reduction of the hydrophobic surface area, while it increases the polar surface area, compared to the other dimerization modes (Figure 5C). This difference is expected to make the helix-mediated dimer more stable. Mutational studies have shown that altering the hydrophobic residues of Nt_17_ to negatively charged residues or by serine phosphorylation, strongly affects the aggregation kinetics by slowing down the aggregation (Gu et al., 2009; Thakur et al., 2009; Mishra et al., 2012). However, Htt-ex1 aggregation was not prevented by these amino-acid changes. In addition to that, according to the aggregation mechanism investigated by Williamson et al., metastable aggregates start to form *via* Nt_17_ packed cores and the polyQ regions being excluded from these cores. These initial aggregates develop then to amyloid nuclei composed of Nt_17_ headpieces and polyQ tracts (Williamson et al., 2010). Based on our dimer simulation results, this aggregation mechanism appears fully plausible. We further find that longer polyQ tracts are needed for amyloid formation as for shorter polyQ sequences, the Nt_17_ helix extends far into the polyQ region and is resistant against *β*-sheet formation. This observation is in agreement with recent NMR spectroscopy results (Elena-Real et al., 2022).

## 4 Conclusion

In this work, we carried out multiple MD simulations to investigate the monomer and dimer structures of a pathogenic Htt-ex1 protein (Htt-Q_48_) and a non-pathogenic (Htt-Q_23_) counterpart. We found rather different monomer structures for the two proteins. One of the most relevant distinctions is that in Htt-Q_23_ the N-terminal helix involves not only Nt_17_ but also the majority of the polyQ_23_ residues. This can be explained by the rather high helix propensity of glutamine, which comes seventh on the helix propensity scale of the twenty proteinogenic amino acids (Pace and Scholtz, 1998). However, for longer polyQ sequences as in Htt-Q_48_, this propensity is not sufficient and the polyQ tract becomes disordered. Here, it should also be considered that the length of an *α*-helix always results from a competition between *α*-helix folding, unfolding into a random coil and the formation of higher-order tertiary structures, and that this competition leads to naturally favored *α*-helix lengths of 9–17 amino acids (Qin et al., 2013). It is thus fully understandable that with increasing polyQ length, this region becomes disordered. As a result, more intra-protein interactions develop, which in turn can lead to *β*-sheet formation, even though glutamine has only a medium propensity to be in a *β*-sheet (Shingate and Sowdhamini, 2012). Here, we observed an increase in bend/turn conformations across the polyQ and PRD regions of Htt-Q_48_, which yielded compact protein structures involving small intra-protein *β*-sheets. In both proteins, the polyP regions mainly adopted PPII structures. However, in Htt-Q_48_ they were disrupted by kinks, which is in line with the compact Htt-Q_48_ conformations, whereas in Htt-Q_23_ the polyP_11_ and polyP_10_ are straight segments that are aligned antiparallel to each other. As a result, Htt-Q_23_ has an elongated shape resulting from the 25 residue-long N-terminal helix and the PPII-turn-PPII motif. This overall fold appeared rather stable and structural fluctuations mainly derived from the first and last ten residues of Htt-Q_23_.

With regard to dimerization, we found that for both Htt-Q_23_ and Htt-Q_48_ this process involves the N-terminal helix, yet to a larger degree in Htt-Q_23_. Here, the helix-helix interaction further stabilized the helix, especially in its second part that involves residues from the polyQ_23_ region, which explains why this region is resistant against *β*-sheet formation. Moreover, interaction through the amphipathic *α*-helix Nt_17_ headpieces minimizes the hydrophobic surface, making this dimer conformation particularly stable. In Htt-Q_48_, on the other hand, different dimerization modes were observed, of which the one involving the two N-terminal helices is one possibility. However, we expect the Nt_17_ helix mediated dimer to be more stable than the others due to the larger burial of hydrophobic residues. An important aspect of Htt-Q_48_ dimerization is that the disordered polyQ regions are considerably involved in the process, allowing *β*-sheets to form on the long time scale (not simulated here). However, this structural transition is slowed down by visible interactions with the PRD. For both Htt-Q_23_ and Htt-Q_48_ the polyP regions are not involved in the dimerization, though their avoidance of the dimer interaction interface is more pronounced in Htt-Q_23_ due to its particular elongated protein shape, allowing the polyP regions to keep away from it.

Overall, we observed that polyQ extension affects the conformational ensemble of Htt-ex1 already at the monomer level and has a direct impact on dimerization. Our results provide insights into the roles of the flanking domains of polyQ in modulating the conformations and the aggregation pathways, thereby providing explanations for several experimental findings at the atomistic level.

## Data Availability

The raw data supporting the conclusion of this article will be made available by the authors, without undue reservation.
