# Supplementary material for: Comparative molecular dynamics simulations of pathogenic and non-pathogenic huntingtin protein monomers and dimers
Source: Front Mol Biosci. 2023 Apr 10;10:1143353. doi: 10.3389/fmolb.2023.1143353 (PMC10123271; doi:10.3389/fmolb.2023.1143353)
Supplement: Supplementary file 1 [file DataSheet1.pdf]

*Supplementary Material*

# Comparative Molecular Dynamics Simulations of Pathogenic and Nonpathogenic Huntingtin Protein Monomers and Dimers

**Mohammed Khaled** [1], **Birgit Strodel** [1,2,*] **and Abdallah Sayyed-Ahmad** [3,*]

[1]Institute of Biological Information Processing (IBI-7: Structural Biochemistry), Forschungszentrum Jülich, 52425 Jülich, Germany
[2]Institute of Theoretical and Computational Chemistry, Heinrich Heine University Düsseldorf, 40225 Düsseldorf, Germany
[3]Department of Physics, Birzeit University, 71939 Birzeit, Palestine
[*]b.strodel@fz-juelich.de, asayyeda@birzeit.edu

| ADF test | Htt-$Q_{23}$ | | | | |
|---|---|---|---|---|---|
| | $R_g$ | $d_{ee}$ | RMSD | helix | SASA |
| **Test Statistic** | -4.41 | -6.51 | -3.7 | -3.29 | -4.26 |
| **p-Value** | $2.81\times10^{-4}$ | $1.14\times10^{-8}$ | $4.17\times10^{-3}$ | $5.16\times10^{-4}$ | $5.16\times10^{-4}$ |
| **Critical Value 1%** | -3.43 | -3.43 | -3.43 | 3.43 | -3.43 |
| **Critical Value 5%** | -2.86 | -2.86 | -2.86 | -2.86 | -2.86 |
| **Critical Value 10%** | -2.57 | -2.57 | -2.57 | -2.57 | -2.57 |
| | Htt-$Q_{48}$ | | | | |
| | $R_g$ | $d_{ee}$ | RMSD | helix | SASA |
| **Test Statistic** | -6.04 | -4.47 | -8.61 | -6.17 | -5.02 |
| **p-Value** | $1.34\times10^{-7}$ | $2.22\times10^{-4}$ | $6.51\times10^{-14}$ | $6.95\times10^{-8}$ | $2.04\times10^{-5}$ |
| **Critical Value 1%** | -3.43 | -3.43 | -3.43 | -3.43 | -3.43 |
| **Critical Value 5%** | -2.86 | -2.86 | -2.86 | -2.86 | -2.86 |
| **Critical Value 10%** | -2.57 | -2.57 | -2.57 | -2.57 | -2.57 |

Table S1. **The Augmented Dickey-Fuller (ADF) test for the Htt-ex1 monomer simulation data.** The ADF test was conducted as a unit-root test to assess the stationary of the Htt-ex1 monomer simulations. The test was applied to the concatenated time series of various observables: the radius of gyration ($R_g$), end-to-end distance ($d_{ee}$), root mean square deviation (RMSD), $\alpha$-helix content, and solvent accessible surface area (SASA).

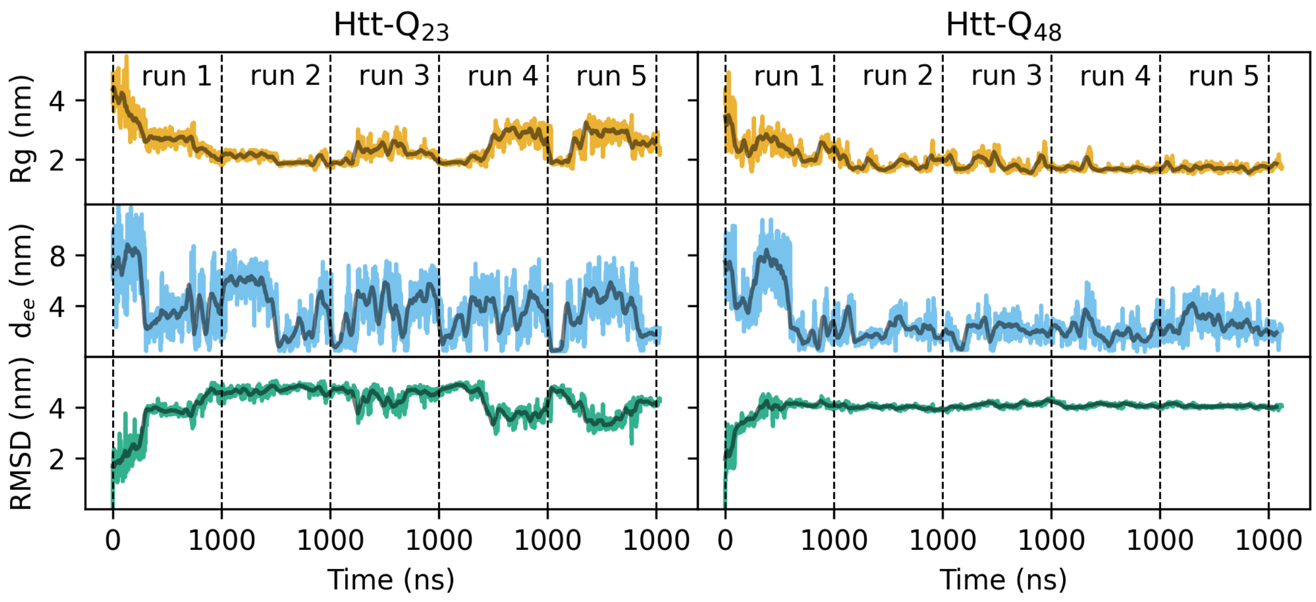


Figure S1: **Results for the** $5 \times 1$ $\mu$**s MD simulations of the Htt-ex1 monomers.** The evolution of $R_g$, RMSD, and $d_{ee}$ for Htt-$Q_{23}$ (left) and Htt-$Q_{48}$ (right) are shown. The thick lines represent 50 ns running averages.

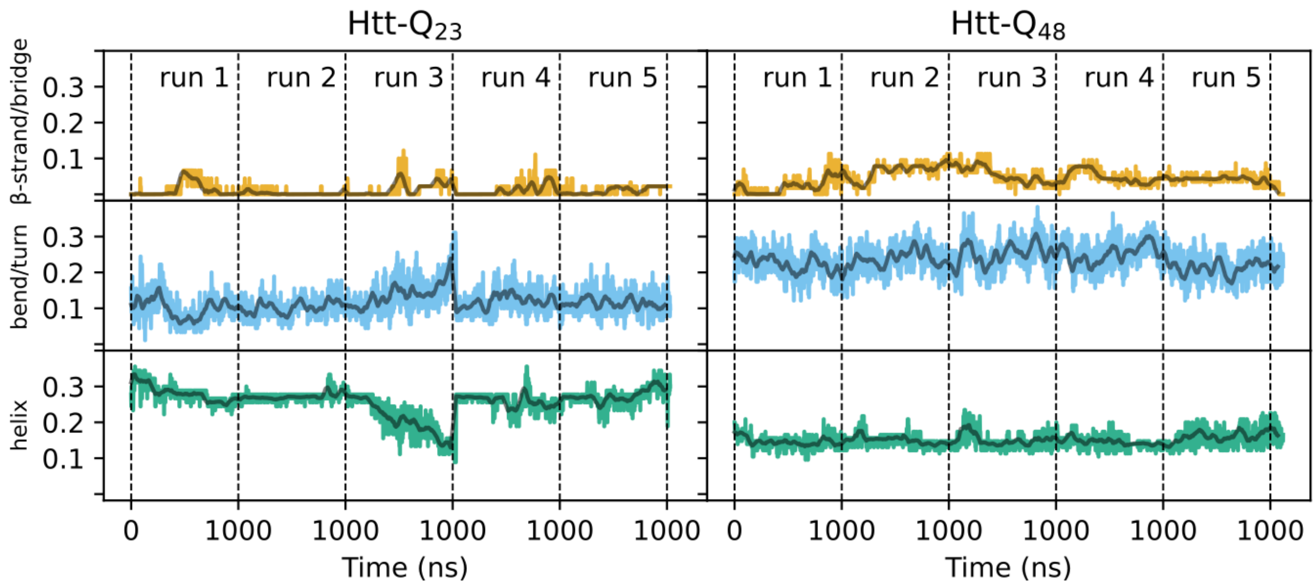


Figure S2: **Secondary structures during the $5 \times 1$ $\mu$s MD simulations of the Htt-ex1 monomers.** The evolution of the $\beta$-strand/bridge, bend/turn, and $\alpha$-helix for Htt-$Q_{23}$ (left) and Htt-$Q_{48}$ (right) are shown. The thick lines represent 50 ns running averages.

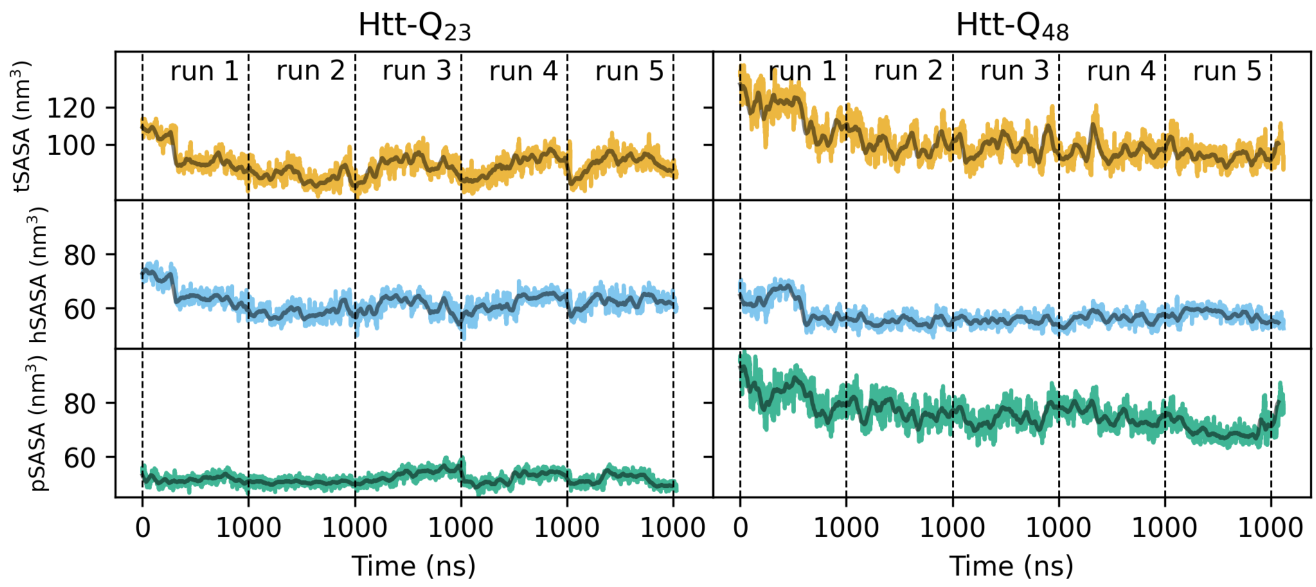


Figure S3: **Solvent accessible surface areas of the Htt-ex1 monomers during the $5 \times 1$ $\mu$s MD simulations.** The evolution of the total SASA (tSASA), the hydrophobic SASA (hSASA), and the polar SASA (pSASA) for Htt-$Q_{23}$ (left) and Htt-$Q_{48}$ (right) are shown. The thick lines represent 50 ns running averages.

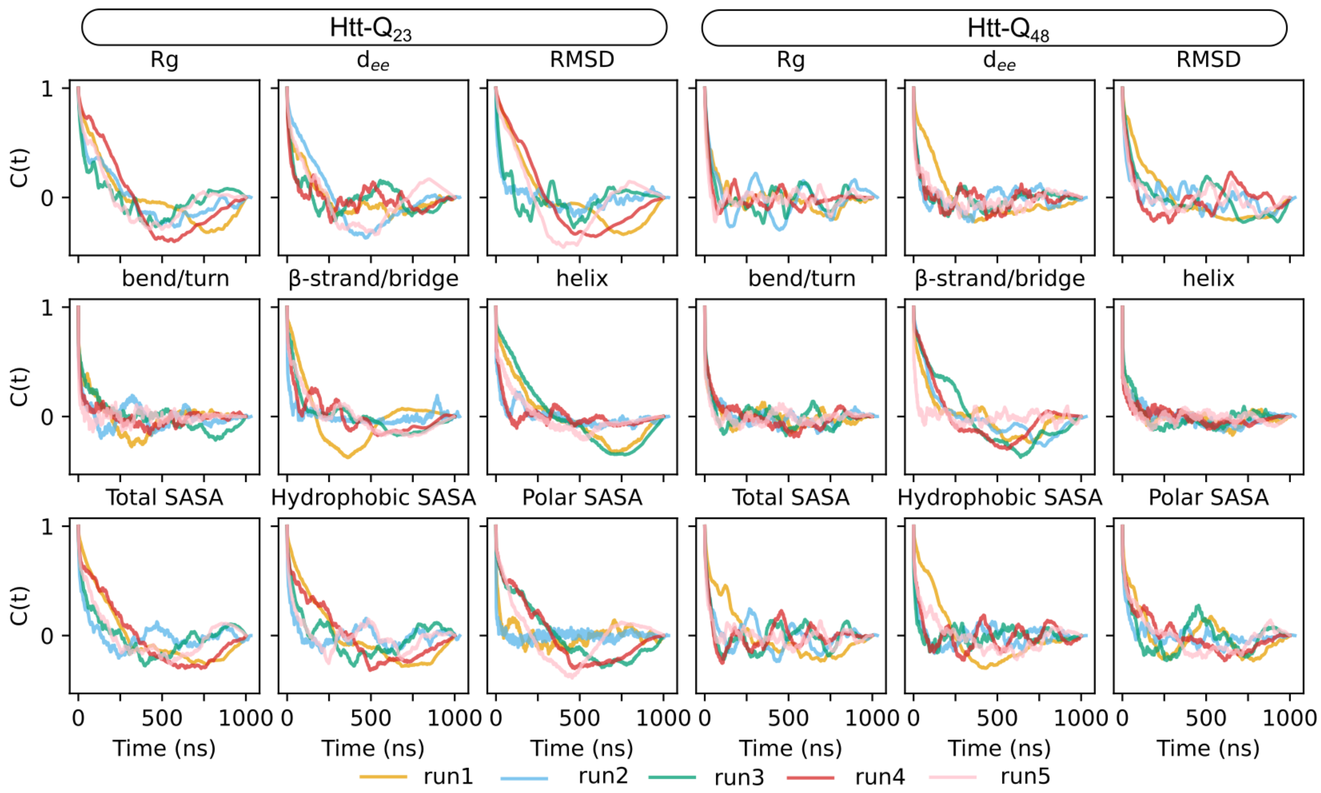


Figure S4: **The autocorrelation functions for a set of structural properties of Htt-ex1 monomers.** The top panels show the autocorrelation functions ($C(t)$) of $R_g$, $d_{ee}$, and RMSD; the middle panels include the $C(t)$ of the content of secondary structures, i.e., bend/turn, $\beta$-strand/bridge, and helix; and the bottom panels display the $C(t)$ of the total, hydrophobic, and polar SASA. The results for Htt-$Q_{23}$ are shown on the left and those for Htt-$Q_{48}$ on the right. All autocorrelations vanish within 100-500 ns, indicating loss of memory from initial structures.

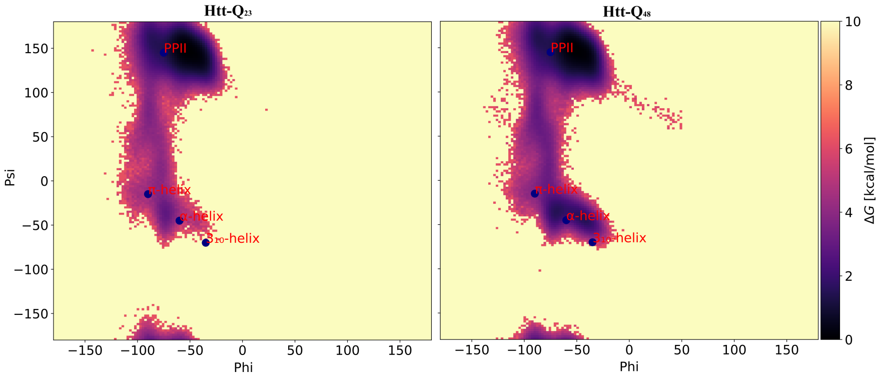


Figure S5: **Ramachandran maps for the polyP regions of Htt-$Q_{23}$ (left) and Htt-$Q_{48}$ (right) monomers.** The color bar shows the value of the free energy ($\Delta$G) in kcal/mol. The blue dots indicate the ideal location of the corresponding secondary structure conformations.

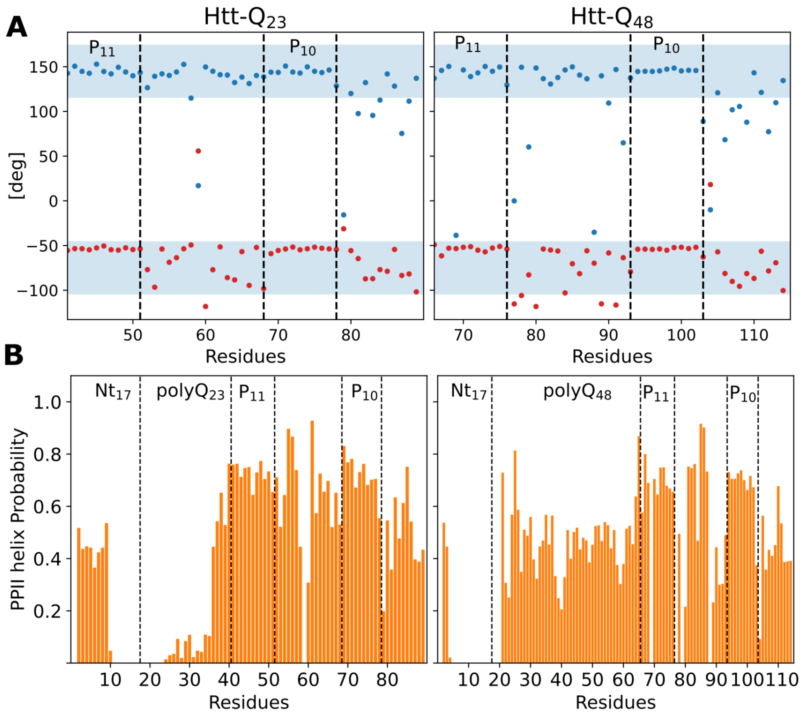


Figure S6: **PPII-helix formation within the Htt-ex1 monomers.** **(A)** The residue-resolved average $\phi$ (red) and $\psi$ (blue) dihedral angles of the PRD region of Htt-$Q_{23}$ (left) and Htt-$Q_{48}$ (right) monomers. The shaded areas indicate the region that defines the PPII helix as in the DSSP-PPII algorithm. **(B)** The PPII-helix propensity of the residues of Htt-$Q_{23}$ (left) and Htt-$Q_{48}$ (right) monomers calculated from the $\phi$ and $\psi$ dihedral angle distributions during the simulations.

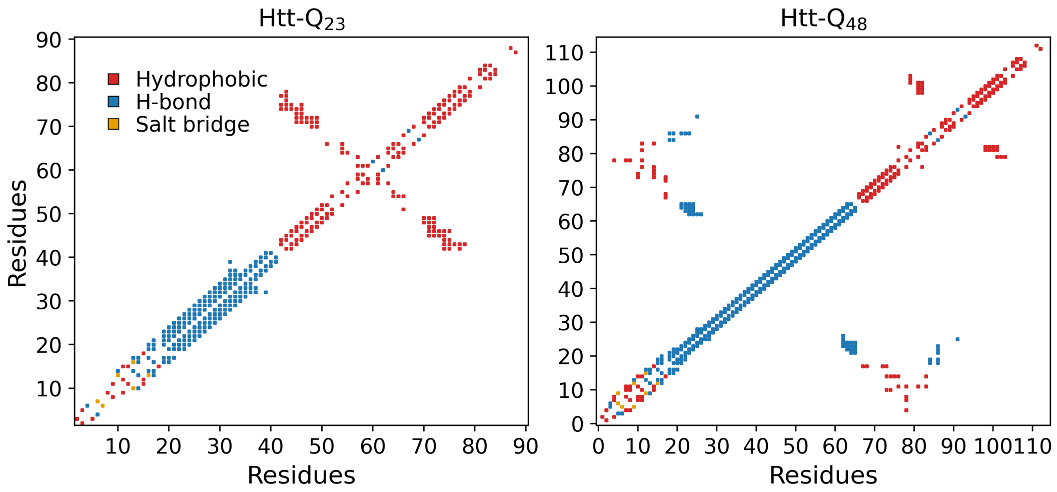


Figure S7: **Types of intra-protein interactions in the Htt-ex1 monomers.** The intra-protein contact maps are colored based on the type of interaction for Htt-$Q_{23}$ (left) and Htt-$Q_{48}$ (right), using red for hydrophobic interactions, blue for H-bonds, and orange for salt bridges.

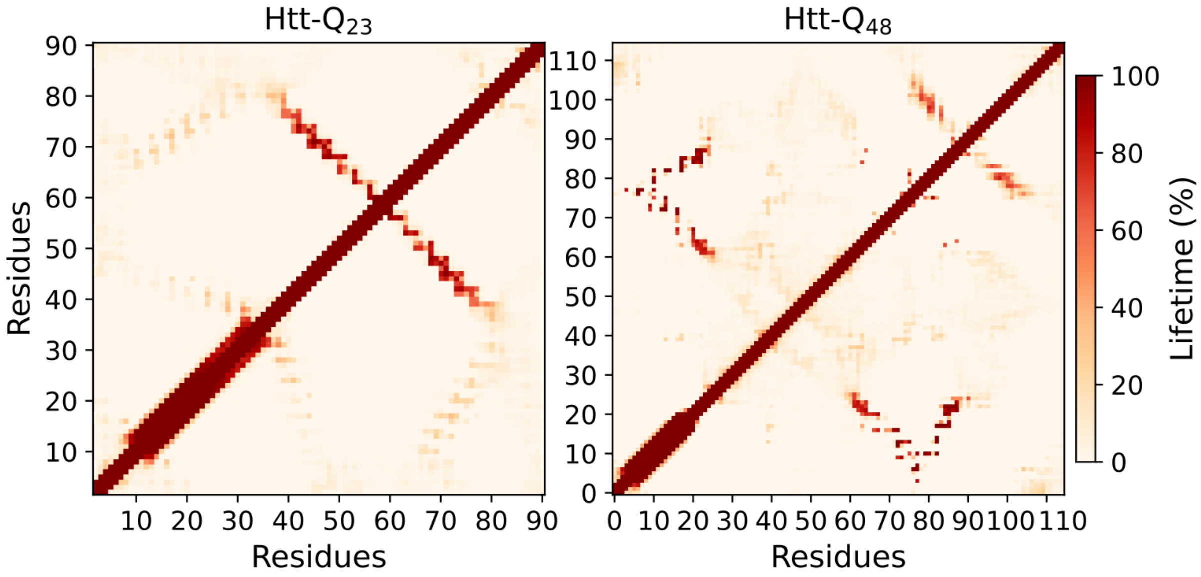


Figure S8: **The lifetime probability of the intra-protein interactions in the Htt-ex1 monomers.** The percent lifetimes for Htt-$Q_{23}$ (left) and Htt-$Q_{48}$ (right) are colored based on the scale on the right.

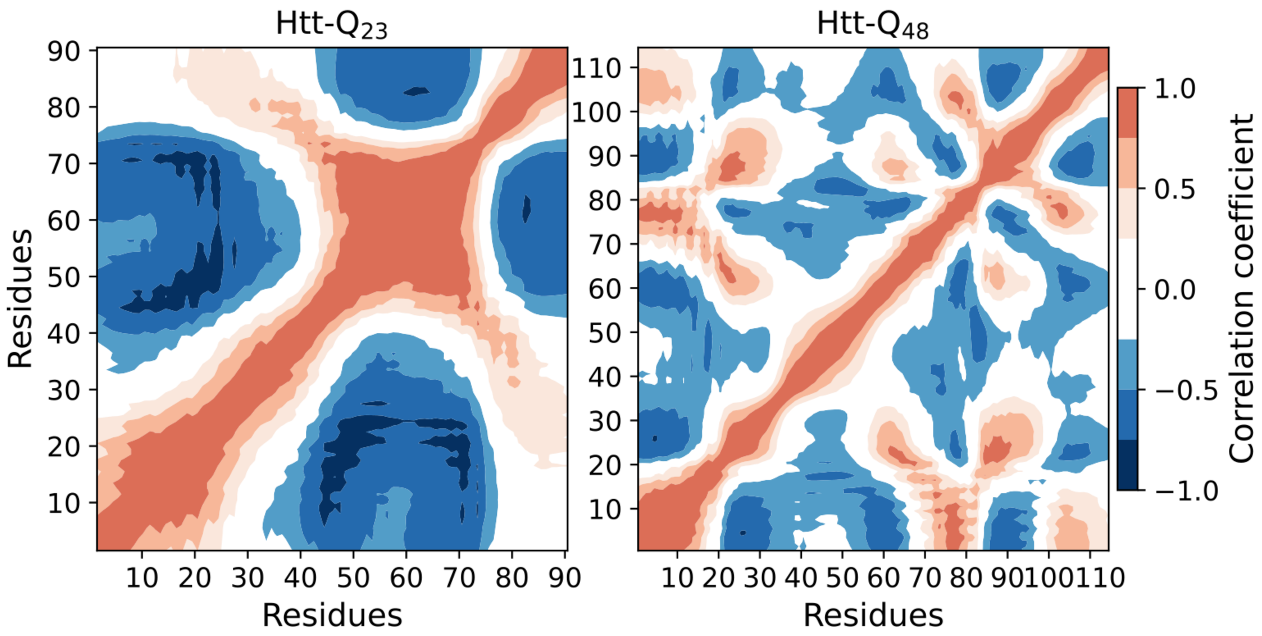


Figure S9: **Dynamic cross-correlation maps of the Htt-ex1 monomers.** Positively and negatively correlated motions are represented in red and blue, respectively, for Htt-$Q_{23}$ (left) and Htt-$Q_{48}$ (right).

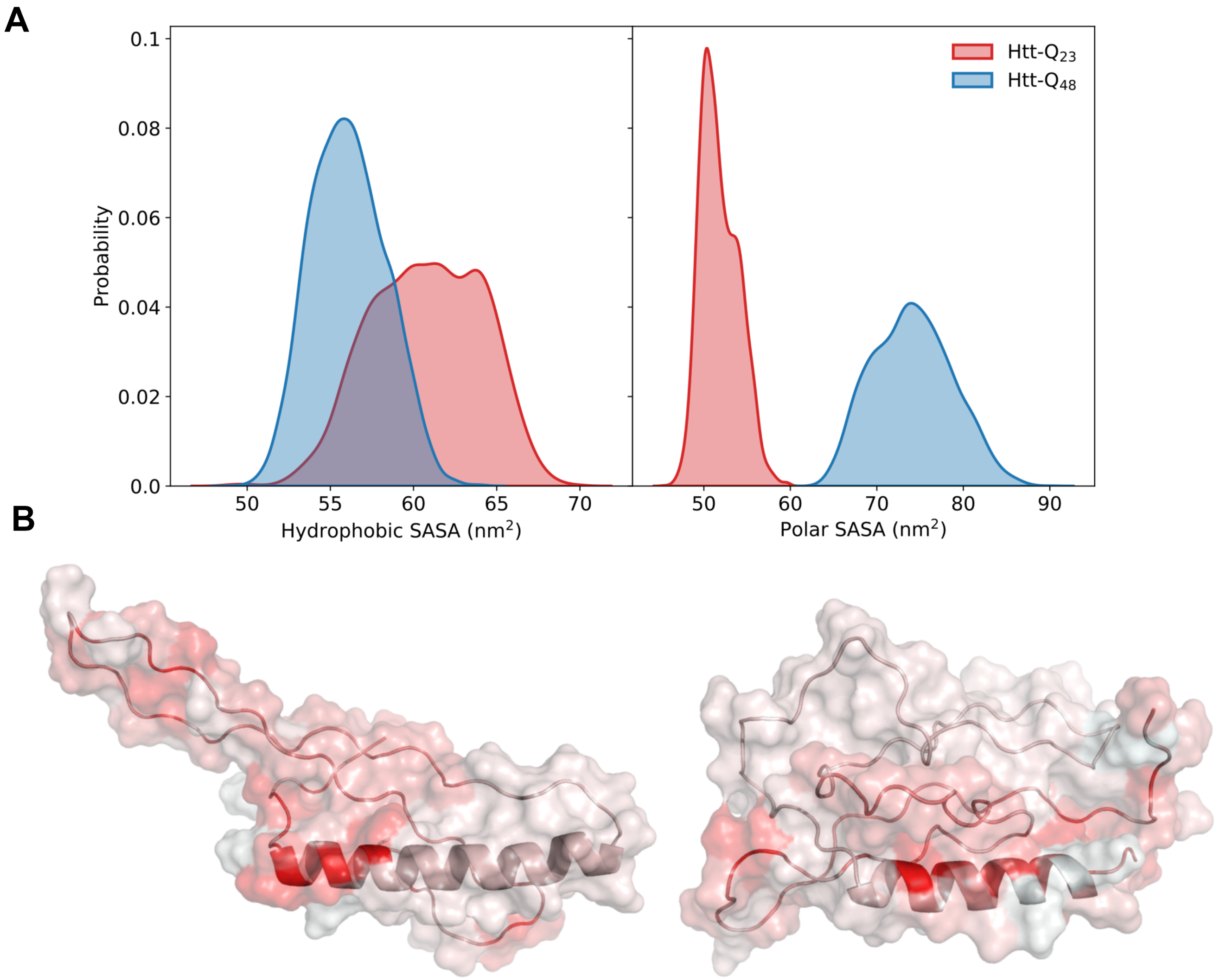


Figure S10: **Solvent accessible surface areas and hydrophobicity of the Htt-ex1 monomers. (A)** The hydrophobic (left) and polar (right) SASA distribution for Htt-$Q_{23}$ (red) and Htt-$Q_{48}$ (blue). **(B)** The most populated cluster structures for Htt-$Q_{23}$ (left) and Htt-$Q_{48}$ (right) colored according their hydrophobicity. The proteins are shown as cartoon plus their van der Waals surface (in transparent). The color represents the hydrophobicity with colors changing from white (not hydrophobic) to red for the amino acids of highest hydrophobicity.

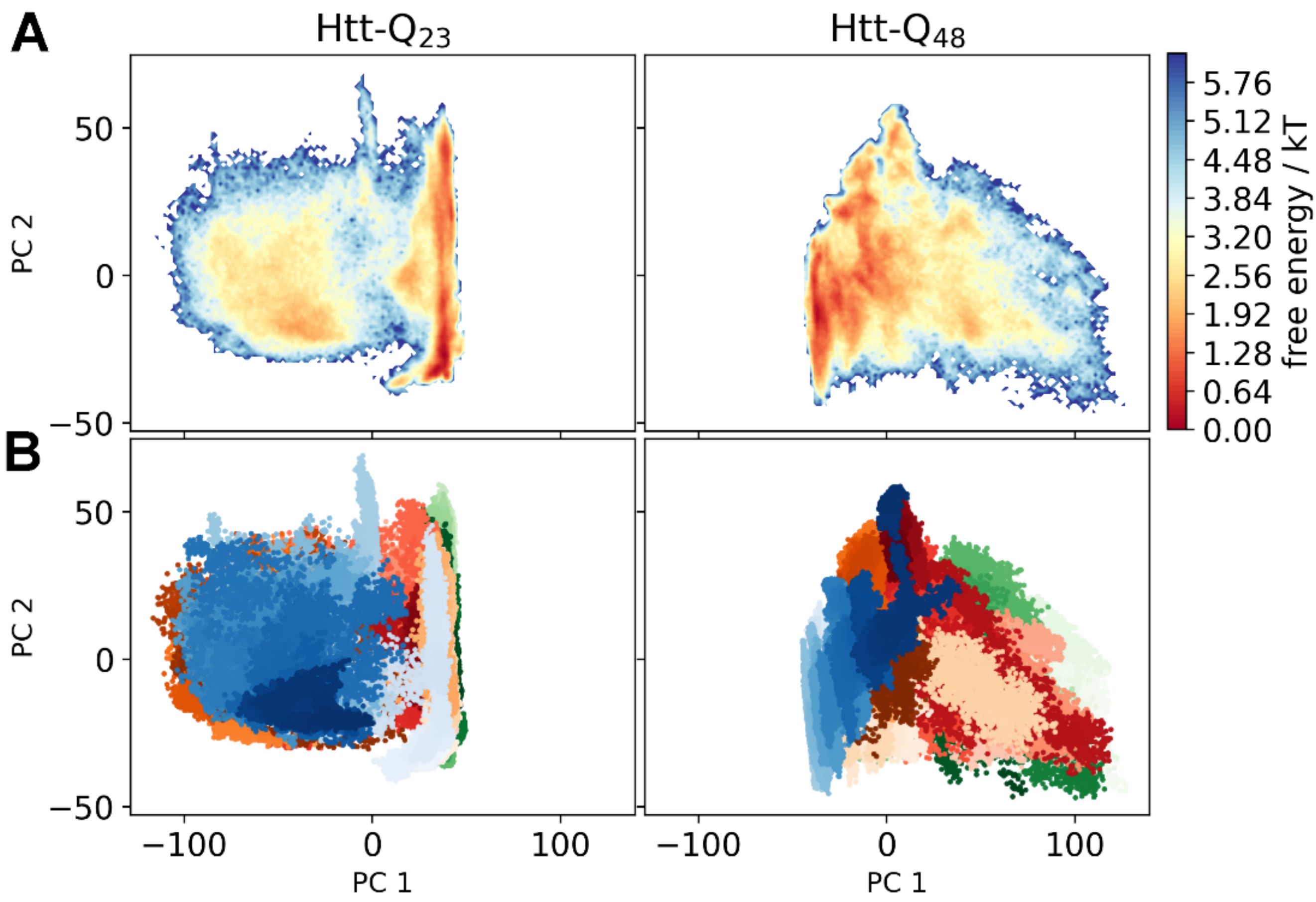


Figure S11: **Principal component analysis for the monomers of Htt-$Q_{23}$ (left) and Htt-$Q_{48}$ (right).** **(A)** The free energy surface as a function of the first two PCs. **(B)** Projection of individual trajectories onto their joint PC1-PC2 space. The color scale represents the space coverage of the individual trajectories (run 2, green; run 3, red; run 4, orange; run 5, blue), showing i) the conformational overlap between the individual trajectories and ii) that also trajectory-specific conformations were sampled.

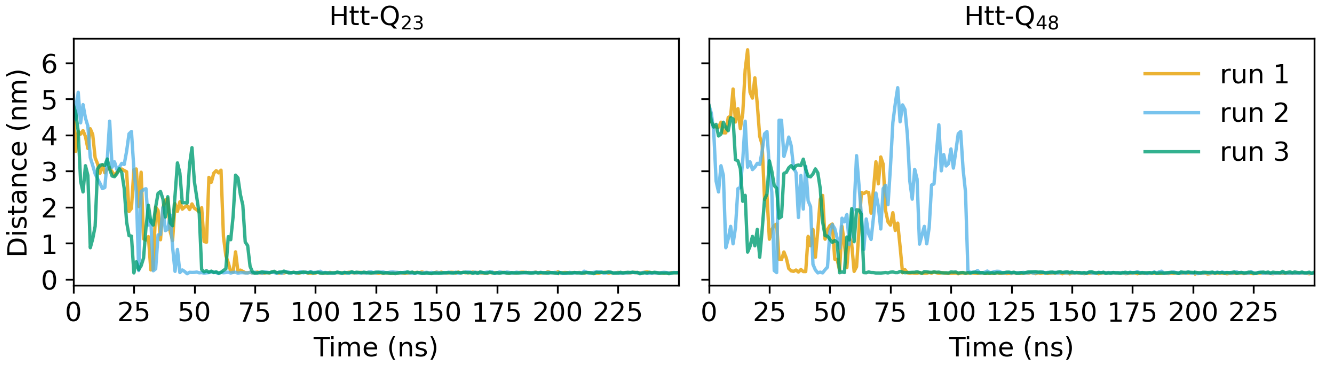


Figure S12: **The minimum distance between the two proteins in the dimer simulations.** The evolution of the inter-protein minimum distance during the three dimer simulations of Htt-$Q_{23}$ (left) and Htt-$Q_{48}$ (right).
